# Effects of Yeast Products on the Apparent Total Tract Macronutrient Digestibility, Oxidative Stress Markers, Skin Measures, and Fecal Characteristics and Microbiota Populations of Healthy Adult Dogs

**DOI:** 10.3390/ani15071046

**Published:** 2025-04-04

**Authors:** Vanessa M. De La Guardia Hidrogo, Lindsey M. Rummell, Kelly S. Swanson

**Affiliations:** 1 Department of Animal Sciences, University of Illinois Urbana-Champaign, Urbana, IL 61801, USA; 2 Wilbur-Ellis Nutrition, Buhl, ID 83316, USA; 3 Division of Nutritional Sciences, University of Illinois Urbana-Champaign, Urbana, IL 61801, USA; 4 Department of Veterinary Clinical Medicine, University of Illinois Urbana-Champaign, Urbana, IL 61801, USA

**Keywords:** brewer’s dried yeast, canine nutrition, canola meal, transport stress

## Abstract

Brewer’s yeast has high nutritional value and contains bioactive compounds that may promote health. Functionalized canola meal (FCM) is a high-fiber ingredient that has been proposed as a carrier for brewer’s yeast. The objective of this experiment was to determine the apparent total tract digestibility (ATTD) of diets containing yeast-enriched FCM and test their effects on the fecal characteristics and microbiota, skin measures, and serum oxidative stress markers of adult dogs. Twelve dogs were fed four extruded kibble diets (control (no FCM or yeast), FCM + low yeast dose, FCM + medium yeast dose, and FCM + high yeast dose) in a replicated 4x4 LSD. Dogs fed yeast-enriched FCM had greater wet fecal output (~15% greater than control). The ATTD of DM, OM, and fat was lower in the diets containing FCM, although the values remained >80% for all macronutrients. The yeast-enriched FCM altered the relative abundance of a few bacterial genera (*Eubacterium brachy*, *Peptoclostridium*, *Ruminococcus gnavus*) and fecal phenol and indole concentrations. Other fecal characteristics, metabolites, bacterial diversity indices, skin measures, or oxidative stress markers were not affected. These findings suggest that yeast-enriched FCM can be incorporated into canine diets without compromising stool quality or nutrient digestibility and may affect microbial metabolism.

## Simple Summary

Yeast-based ingredients are highly nutritious and contain bioactive compounds that may support gut health. Functionalized canola meal (FCM) is a fiber-rich ingredient that may be used in combination with yeast-based ingredients to provide multiple benefits. In this experiment, FCM enriched with varying levels of brewers dried yeast was incorporated into kibble diets for dogs. The objective was to measure the nutrient digestibility of yeast-enriched FCM diets and to test their effects on the stool characteristics, microbiota composition, skin health measures, and oxidative stress markers of healthy adult dogs. The digestibility rates of all macronutrients and energy were above 80% but the dry matter, organic matter, and fat digestibility rates were lower in diets containing the yeast-enriched FCM. The dogs fed diets containing yeast-enriched FCM also had a greater fecal output (~15% greater than control). The yeast-enriched FCM altered the relative abundance of a few bacterial genera (*Eubacterium brachy*, *Peptoclostridium*, *Ruminococcus gnavus*) and fecal phenol and indole concentrations. The skin health measures and oxidative stress markers were not affected by the treatment. Our findings suggest that yeast-enriched FCM can be safely added to canine diets without compromising stool quality or nutrient digestibility and may provide benefits to gastrointestinal health, although more research is necessary to optimize the dosages, mixtures, or both.

## 1. Introduction

Functional ingredients are added to pet foods to support wellness by offering health advantages that extend beyond meeting basic nutritional needs [1]. Brewer’s yeast, a by-product of the brewing industry, is a valuable source of high-quality protein, dietary fiber, B-complex vitamins, and minerals [2]. Additionally, brewer’s yeast can adsorb phenolic acids, enhancing its antioxidant activity and nutritional value [3,4]. Recent studies have demonstrated the functional properties of yeast and its constituents in respect to the gut microbiota, immune response, and oxidative stress among various species [5,6]. In dogs, yeast-derived polyphenols, mannanoligosaccharides, and β-glucans have been shown to modulate the gut microbiota, enhance the immune response, and reduce oxidative stress and the inflammatory response [7,8,9,10]. However, due to variations in yeast strain, fermentation conditions, and processing techniques, the safety and optimal feeding dosages of each product must be evaluated in the target species (e.g., dogs, cats).

While many yeast-based ingredients are incorporated into diets individually, others are combined with carriers to facilitate handling and ensure uniform distribution within the diet mixture. Recently, functionalized canola meal (FCM) has been proposed as an active carrier for dried brewer’s yeast. The FCM is a nutrient-dense ingredient, containing approximately 45% protein and 35% total dietary fiber (TDF), along with dietary polyphenols such as sinapic acid, protocatechuic acid, syringaldehyde, hydroxybenzoic acids, and hydroxycinnamic acids [11]. Although this blend has been used commercially, limited data are available regarding its effects on canine gut health outcomes.

A previous experiment conducted in our laboratory determined the apparent total tract digestibility (ATTD) of diets top-dressed with yeast-enriched FCM and their effects on the fecal quality, metabolite concentrations, microbiota populations, and immune function of healthy adult dogs [7]. The results of that experiment demonstrated no adverse effects, although further research is needed to optimize the dosing and evaluate the functionality of the yeast-enriched FCM when included as an ingredient in an extruded kibble diet. Additionally, polyphenols present in the brewer’s yeast and FCM blend may exert antioxidant effects, including free radical scavenging and the upregulation of endogenous antioxidant enzymes [12]. These mechanisms could help mitigate oxidative damage, which compromises the cellular integrity and function, and may potentially enhance the skin barrier [13]. Therefore, the objective of this experiment was to evaluate the effects of extruded diets supplemented with FCM enriched with different levels of dried yeast on different health parameters of adult dogs. The parameters evaluated included (1) the ATTD of macronutrients and energy; (2) fecal characteristics, metabolites, and microbiota populations; (3) skin integrity measures; and (4) serum oxidative stress markers after a transport stress challenge. We hypothesized that extruded diets containing yeast-enriched FCM would maintain the ATTD of macronutrients and energy and fecal characteristics, while improving skin integrity and promoting beneficial changes in fecal microbiota populations and metabolites. Finally, we hypothesized that dogs supplemented with yeast-enriched FCM would have reduced markers of oxidative stress when subjected to transport stress. 

## 2. Materials and Methods

### 2.1. Animals and Housing

Twelve adult female beagle dogs (body weight = 9.75 ± 0.83 kg; body condition score = 6.1 ± 0.7; age = 6.2 ± 1.6 yr) were used. All dogs were individually housed in pens approximately 1.2 m wide × 2.4 m long in an environmentally controlled room, maintained on a 12 h light/12 h dark cycle in the Veterinary Medicine Basic Sciences Building at the University of Illinois. The dogs were provided with toys for behavioral enrichment and socialized regularly in compatible groups. The dogs had ad libitum access to fresh water at all times and were fed once a day. The amount of food offered was adjusted on a weekly basis to maintain body weight. The body weights and body condition scores [14] were assessed once a week prior to feeding.

### 2.2. Experimental Design, Diet, and Treatments

The dogs were used in a replicated 4 × 4 Latin square design with four 35 d periods (n = 12/group). Each period consisted of a 25 d diet transition phase followed by 5 d for fecal sample collection and 5 d for blood sample collection, transport stress testing, and skin measurements. Four extruded diets formulated to meet all of the essential nutrients recommended by the Association of American Feed Control Officials [15] for adult dogs at maintenance were fed: (1) control diet containing no FCM or yeast; (2) diet containing FCM + low yeast dose (LY); (3) diet containing FCM + medium yeast dose (MY); (4) diet containing FCM + high yeast dose (HY). Dogs were randomly assigned to each dietary treatment, and the diets were coded so that the researchers were blinded to the treatment identities throughout the experiment.

### 2.3. Fecal Sample Collection and Fecal Characteristics

Following the 25 d diet transition phase, all fecal matter was collected over the next 5 d. All feces voided were collected from the floor of the pen, scored, weighed, and stored at −20 °C until the nutrient digestibility analysis. All fecal scores were assigned using the following scale: 1 = hard, dry pellets, small hard mass; 2 = hard, formed, dry stool; remains firm and soft; 3 = soft, formed, and moist stool, retains shape; 4 = soft, unformed stool, assumes shape of container; 5 = watery liquid that can be poured.

On d 26 of each experimental period, fresh fecal samples were collected within 15 min of defecation. The fecal pH was measured immediately using an AP10 pH Meter (Denver Instrument, Bohemia, NY, USA) equipped with a Beckman Electrode (Beckman Instruments Inc., Fullerton, CA, USA). The remaining samples were aliquoted for determination of the fecal dry matter (DM), metabolites (short-chain fatty acids (SCFA), branched-chain fatty acids (BCFA), phenols, indoles, and ammonia), and microbiota populations.

### 2.4. Transport Stress and Blood Collections

On day 31 of each experimental period, the dogs were placed into individual dog carriers, loaded into a van, and transported for 45 min to evaluate the effects of travel stress. Prior to transport, fasted blood samples were collected via jugular puncture. The samples were immediately transferred to vacutainer tubes containing K_2_EDTA (no. 365974 BD Microtainer; Becton Dickinson, Franklin Lakes, NJ, USA) for a hematology analysis and with vacutainer tubes with a clot activator additive and gel for serum separation (no. 368660 BD Vacutainer Plus; Becton Dickinson, Franklin Lakes, NJ, USA) for the serum chemistry and oxidative stress marker analysis. Post-transport (3 h), additional blood samples were collected into vacutainer tubes for the oxidative stress marker analysis.

After collection, the blood samples were centrifuged at 1300× *g* at 4 °C for 10 min using a Beckman CS-6R centrifuge (Beckman Coulter Inc., Brea, CA, USA) for serum separation. Once the serum was harvested, it was transported to the University of Illinois Veterinary Medicine Diagnostics Laboratory for a serum chemistry analysis using a Hitachi 911 clinical chemistry analyzer (Roche Diagnostics, Indianapolis, IN, USA). The K_2_EDTA tubes, containing uncoagulated blood, were cooled (but not frozen) and transported to the University of Illinois Veterinary Medicine Diagnostics Laboratory for hematology analyses. The serum concentrations of oxidative stress markers, namely malondialdehyde (MDA) and superoxide dismutase (SOD), were measured using commercial ELISA kits (MDA: MBS2605193, MyBioSource, San Diego, CA, USA; SOD: MBS2104718, MyBioSource).

### 2.5. Skin Measures

On day 35 of each experimental period, the transepidermal water loss (TEWL; Tewameter TM 300 MDD, Courage + Khazaka Electronic GmbH, Cologne, Germany), hydration status (Corneometer CM 825, Courage + Khazaka Electronic GmbH), and sebum concentrations (external Sebumeter SM 815, Courage + Khazaka Electronic GmbH) were measured. To prevent hair interference and ensure reading accuracy, all dogs underwent shaving in the lumbar, inguinal, and inner ear regions 24 h prior to the assessments. All assessments of skin parameters were conducted in the same environmentally controlled room throughout the experiment.

### 2.6. Chemical Analysis and Macronutrient and Energy Digestibility Calculations

The diet and total fecal samples were analyzed for DM and ash (Methods 934.01 and 942.05) according to the Association of Official Analytical Chemists guidelines [16], with the organic matter (OM) calculated. The crude protein levels of the diets and feces were calculated from Leco total nitrogen values (TruMac N, Leco Corporation, St. Joseph, MI, USA) in accordance with the AOAC (method 992.15) [10]. The total lipid content (acid-hydrolyzed fat; AHF) was determined according to the methods of the American Association of Cereal Chemists [17] and Budde [18]. The gross energy (GE) was measured using an oxygen bomb calorimeter (model 6200, Parr Instruments, Moline, IL, USA). Subsequently, the ATTD values for macronutrients and energy were calculated using the following equation: digestibility (%) = [nutrient intake (g/d) − fecal output (g/d)]/nutrient intake (g/d) × 100%.

### 2.7. Fecal Metabolite Analysis

The fecal SCFA concentrations were determined via gas chromatography as described by Erwin et al. [19]. The analysis was performed on a Hewlett-Packard 5890A series II gas chromatograph equipped with a glass column (180 cm × 4 mm i.d.) packed with 10% SP-1200/1% H_3_PO_4_ on 80/100+ mesh Chromosorb W-AW (Supelco Inc., Bellefonte, PA, USA). Nitrogen gas served as the carrier at a flow rate of 75 mL/min. The oven, detector, and injector temperatures were set at 125, 175, and 180 °C, respectively. The phenol and indole concentrations were determined using gas chromatography following the methods described by Flickinger et al. [20], while the fecal ammonia concentrations were determined as per Chaney and Marbach’s study [21].

### 2.8. Fecal Microbiota Analysis

The fecal DNA was extracted using DNeasy PowerLyzer PowerSoil kits (Qiagen, Carlsbad, CA, USA), with its concentration measured using a Qubit 3.0 Fluorometer (Life Technologies, Carlsbad, CA, USA). The DNA quality was evaluated via agarose gel electrophoresis (E-Gel EX Gel 1%; Invitrogen, Carlsbad, CA, USA). The 16S rRNA gene amplicons were generated using a Fluidigm Access Array (Fluidigm Corporation, South San Francisco, CA, USA) in combination with a Roche High-Fidelity Fast Start Kit (Roche, Indianapolis, IN, USA). The primers 515F (5′-GTGCCAGCMGCCGCGGTAA-3′) and 806R (5′-GGACTACHVGGGTWTCTAAT-3′), target a 252 bp fragment of the V4 region of the 16S rRNA gene, were used for amplification (primers synthesized by IDT Corp., Coralville, IA, USA) [22]. The CS1 forward tags and CS2 reverse tags were added according to the Fluidigm protocol. The quality of the amplicons was assessed using a fragment analyzer (Advanced Analytics, Ames, IA, USA) to confirm the amplicon regions and sizes. A DNA pool was generated by combining equimolar amounts of the amplicons from each sample. The pooled samples were then size-selected on a 1–2% agarose E-gel (Life Technologies, Grand Island, NY, USA) and extracted using a Qiagen gel purification kit (Qiagen, Valencia, CA, USA). The cleaned size-selected pooled products were run on an Agilent Bioanalyzer to confirm the appropriate profile and average size. Illumina sequencing was performed on a MiSeq using V3 reagents (Illumina Inc., San Diego, CA, USA) at the Roy J. Carver Biotechnology Center at the University of Illinois.

### 2.9. Bioinformatics

The forward reads were trimmed using the FASTX-Toolkit and the resulting sequences were processed using the QIIME 2.0 software pipeline [23]. Sequences with a quality score ≥20 were filtered for quality control with DADA2 [24]. The samples were rarefied to an even depth and the taxonomy was assigned using the Naive Bayes classifiers trained on the Silva database (v.138) [25,26,27]. Diversity analyses were performed via the q2-diversity plugin, and the β-diversity was calculated using the weighted and unweighted UniFrac distances [28]. Visualization of the UniFrac distances was achieved using principal coordinate analysis (PCoA) plots.

### 2.10. Statistical Analyses

The data were analyzed using PROC UNIVARIATE to test the normality. All non-normal data were transformed to meet the assumptions of equal variance and normality of the residuals. Subsequently, the data were analyzed using the PROC MIXED procedure. A statistical analysis evaluated the different levels of dietary treatment as the main effect with the random effect of the dog. Statistical differences were determined using a Fisher-protected least significant difference test with a Tukey adjustment to control for experiment-wise error. Orthogonal contrasts were used to examine the differences between the control and FCM treatment groups. The fecal scores were analyzed using PROC FREQ with the Fisher’s exact test to evaluate the differences in the relative frequency of fecal scores among the treatment groups. All data were analyzed using the Statistical Analysis Systems (SAS) software version 9.4 (SAS Institute, Inc., Cary, NC, USA). Significance was declared at *p* ≤ 0.05, and trends reported if 0.05 < *p* ≤ 0.10.

## 3. Results

Among the 12 dogs initially enrolled in the experiment, one dog was removed due to a diagnosed renal condition unrelated to the treatments. Therefore, only 11 dogs were included in the statistical analysis. No other health complications were observed during the experiment.

The analyzed chemical compositions and energy contents of the diets are presented in Table 1. All diets had similar macronutrient compositions, with small differences in their protein and fiber contents. The average food intake was 147.6 g/d, which did not differ among the treatments (Table 2). However, the orthogonal contrast analysis revealed higher as-is intake in dogs fed the FCM-supplemented diets relative to the control (*p* = 0.05). The fecal output, expressed on an as-is and DM basis, tended to be lower (*p* = 0.07) in dogs fed the control diet than all other diets. Similarly, the orthogonal contrast analysis showed that the fecal output was greater (*p* = 0.01) when the dogs were fed the yeast-enriched FCM diets vs. the control. All diets were highly digestible, with the ATTD being >80% for all macronutrients and for total energy. The ATTD values of DM, OM, CP, and GE were not different among treatments. However, the LY and HY diets tended to have lower (*p* = 0.06) ATTD values for fat than MY or CTRL. The orthogonal contrast analysis showed that during supplementation with the yeast-enriched FCM, the dogs had lower DM, OM, and fat digestibility rates vs. the control.

The fecal DM and pH values were not different among the diets (Table 3). The fecal pH values tended to be lower (*p* = 0.10) in the control diet than the yeast-enriched FCM-supplemented diets. No differences (*p* = 0.86) were observed in the relative frequency rates of fecal scores (1 to 5 scale; Supplementary Figure S1). The most frequently observed fecal score among the treatments was 3.0, which was characterized as a moist stool that retains its shape. The concentrations of fecal SCFA and BCFA did not differ among the treatment groups. However, the fecal indole and total phenol + indole concentrations were higher (*p* = 0.03) in the dogs fed the MY diet than those fed the LY diet. No differences were observed for the fecal phenol or ammonia concentrations among the treatment groups. The orthogonal contrast analysis showed that the dogs tended to have lower (*p* = 0.09) phenol concentrations vs. the control.

The hematology and serum metabolite profiles are presented in Table 4 and Table 5, respectively. Irrespective of the treatment, the mean corpuscular hemoglobin concentrations and white blood cell concentrations were below the reference range for adult dogs. None of the hematology measurements were different among the treatment groups. However, the orthogonal contrast analysis showed that the eosinophil (*p* = 0.03) and basophil (*p* = 0.02) concentrations were lower in the dogs fed yeast-enriched FCM vs. the control. All mean serum metabolite concentrations were within the standard reference range for adult dogs, aside from the albumin/globulin ratio, which was slightly elevated for all dogs. The serum blood urea nitrogen concentrations were greater (*p <* 0.01) in the dogs fed the control and MY diets than those fed the HY diet. Additionally, the dogs fed the control diet tended to have greater chloride (*p* = 0.07) and corticosteroid isozyme of alkaline phosphatase (*p =* 0.09) concentrations. The orthogonal contrast analysis showed that the dogs fed the yeast-enriched FCM tended to have lower BUN (*p =* 0.09) and sodium (*p =* 0.09) concentrations, as well as reduced potassium (*p =* 0.03) and chloride (*p <* 0.01) concentrations in their serum.

The serum SOD and MDA concentrations collected before and after transport stress are reported in Table 6. An increase (*p* < 0.01) in serum MDA concentrations was observed after transport. Likewise, the serum SOD concentrations tended to increase after transport (*p* = 0.07). The serum SOD and MDA concentrations were unaffected by treatment group, however. The skin measurement data are presented in Table 7. The skin sebum concentration, hydration status, and TEWL values were not affected in the back, inguinal, and ear regions among the treatment groups.

**Figure 1:**
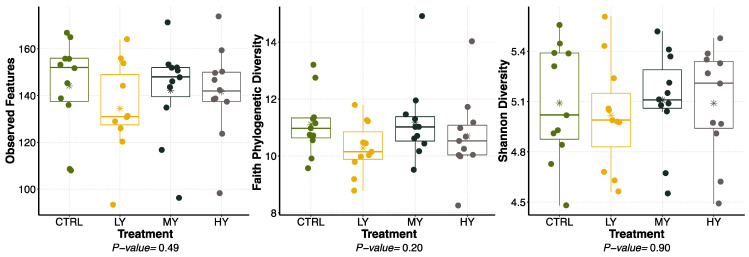
Bacterial alpha diversity measures of fecal samples collected from dogs fed extruded diets containing FCM enriched with different levels of dried yeast. Alpha diversity is represented by observed features, Faith’s phylogenetic diversity, and the Shannon Diversity Index. CTRL = control, LY = FCM + low yeast dose, MY = FCM + medium yeast dose, HY = FCM + high yeast dose. * Denote average values for each treatment.

**Figure 2:**
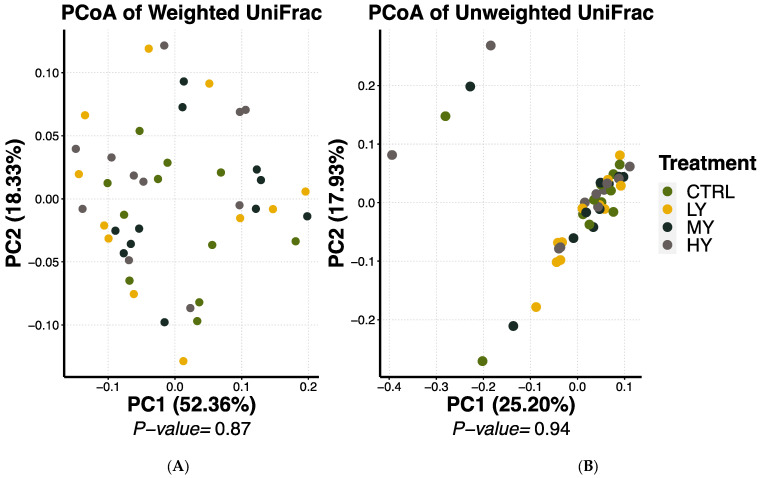
Bacterial beta diversity rates of dogs fed extruded diets containing FCM enriched with different levels of dried yeast: weighted (**A**) and unweighted (**B**) PCoA plots showing similar fecal microbial community structures among treatments. CTRL = control, LY = FCM + low yeast dose, MY = FCM + medium yeast dose, HY = FCM + high yeast dose.

No differences in bacterial alpha diversity measures were observed in the feces of the dogs (Figure 1). Similarly, the bacterial beta diversity analysis results for the fecal samples were not different among the treatments when the weighted (quantitative) and unweighted (qualitative) UniFrac distances of the microbial communities were analyzed (Figure 2). The predominant bacterial phyla present in the fecal samples were *Firmicutes* (61.45%), *Fusobacteriota* (17.32%), and *Bacteroidota* (13.99%). Over 20 fecal bacterial genera were detected with a relative abundance rate ≥ 1% (Table 8). The most abundant bacterial genus was *Fusobacterium*, with an average relative abundance rate of 17.32% across all treatments. The other major bacterial genera (relative abundance > 5%) were *Lactobacillus* (10.96%), *Peptoclostridium* (10.83%), *Bacteroides* (7.46%), and *Allobaculum* (6.54%). The relative abundance of fecal the *Eubacterium brachy* group was lower (*p* < 0.05) in the dogs fed the LY diet than those fed the HY and MY diets. The relative abundance levels of the fecal *Ruminococcus gnavus* group (*p* = 0.09) and *Peptoclostridium* (*p* = 0.10) also tended to differ among the treatments. The orthogonal contrast analysis showed that the dogs supplemented with the yeast-enriched FCM had lower fecal abundance levels of the *Ruminococcus gnavus* group (*p* = 0.04) and *Peptoclostridium* (*p* = 0.02) vs. the control.

## 4. Discussion

Limited research exists on the functional properties of yeast-enriched FCM in companion animal diets. In a previous experiment, our laboratory determined how yeast-enriched FCM impacted the ATTD of dietary macronutrients and evaluated its effects on the immune function, fecal characteristics, and fecal microbiota of healthy adult dogs [7]. In that experiment, the FCM was dosed by top dressing a complete and balanced kibble diet. To test its application in complete and balanced pet foods, the yeast-enriched FCM was incorporated into the diet mix and subjected to extrusion conditions. Moreover, there has not been any research conducted to test the effects of yeast-enriched FCM on oxidative stress markers and skin health. Therefore, the current experiment was conducted to evaluate the ATTD of macronutrients in diets containing yeast-enriched FCM and to test its effects on the stool quality, fecal metabolite concentrations and microbiota populations, serum oxidative stress markers, and skin health markers of adult dogs.

The chemical compositions of the diets in this experiment were not different, except for a slight increase in TDF in the diets supplemented with yeast-enriched FCM. Although no differences were observed in the overall food intake across treatments, the dogs consuming the yeast-enriched FCM exhibited a tendency for a greater fecal output, with the most pronounced effects observed in dogs fed the LY diet. The FCM is a processed variant of canola meal that preserves its high dietary fiber content (35% TDF) [7]. The fiber present in canola meal is predominantly insoluble, with up to 90% of the non-starch polysaccharides being in that form [29]. Insoluble fibers can mechanically stimulate the lining of the colon, providing a laxative effect [30]. Additionally, yeast cell walls contain soluble fiber (e.g., beta-glucans), which can increase the fecal bulk and stimulate intestinal motility [31,32,33]. Together, these factors likely accelerate the transit rate, increase the fecal bulk, and contribute to the observed increase in fecal output. Despite the differences in fecal output, the subjective fecal scores were similar for all dogs, regardless of treatment.

All ATTD rates were above 80%, which is considered to be adequate in dogs. However, the ATTD rates for DM, OM, and fat were lower in the dogs consuming diets containing the yeast-enriched FCM, likely due to the higher fiber contents of these diets. The results of previous research has demonstrated an inverse relationship between fiber inclusion and the ATTD of macronutrients and energy in dogs [34]. In another experiment, Reilly et al. [35] observed reduced ATTD of DM, OM, acid-hydrolyzed fat, and metabolizable energy in dogs consuming an extruded diet formulated with a 29.88% inclusion of a dried yeast product used as the primary protein source. In that experiment, the dried yeast-containing diet had the highest fiber content (17.7% vs. 11.2% in the control diet), reflecting the macronutrient profile of yeast cells. Dietary fiber can reduce macronutrient and energy digestibility through various mechanisms, such as binding bile acids, which limits fat emulsification, and accelerating the gastrointestinal transit, which reduces the time available for digestion and absorption within the small intestine [27]. Considering the differences observed in ATTD and fecal output rates, supplementation with yeast-enriched FCM may have potential applications in the formulation of weight management diets.

The supplementation of yeast-enriched FCM did not alter the fecal SCFA or BCFA concentrations in the current experiment, agreeing with the results of our previous experiment [7]. As SCFA are rapidly and extensively (~95%) absorbed by the colonocytes of the host, it is often difficult to detect changes in fecal samples [36]. Although the fecal SCFA concentrations were not different among the treatment groups, the dogs consuming the LY diet showed reductions in their indole and total phenol and indole concentrations. This may be attributed to changes in microbial metabolism driven by the dietary composition. Specifically, a greater availability of non-digestible carbohydrates may shift the microbial activity towards saccharolytic fermentation and reduce the production of protein-derived catabolites [37,38]. Another potential explanation may be due to a reduction in tryptophan availability for microbial deamination in the hindgut. Phenols and indoles, produced by the microbial deamination of aromatic amino acids [39], are associated with adverse effects, including fecal malodor, an increased inflammatory response, tissue permeability, and dysbiosis [40,41,42]. While our findings suggest a beneficial modulation of microbial metabolism, microbial indole derivatives can exert a wide range of biological effects in eukaryotic systems, including roles in inflammation, epithelial integrity, and systemic signaling [43]. Therefore, further research is needed to determine the physiological relevance of individual indole compounds and the extent to which the dietary modulation of microbial metabolism may influence host health [44].

In agreement with the results of previous experiments testing different yeast products [45,46,47], yeast-enriched FCM supplementation slightly affected the fecal microbiota. In the current experiment, no differences were observed in alpha and beta diversity metrics. These results indicate that the yeast-enriched FCM had minimal effects on the fecal microbiota composition of healthy adult dogs. Likewise, no significant differences in the relative abundance levels of the main bacterial taxa were observed with yeast-enriched FCM supplementation based on shotgun metagenomic sequencing [11]. In the current experiment, the relative abundance levels of only a few taxa were altered by the yeast-enriched FCM consumption. Among these, the *Eubacterium brachy* group had a lower abundance in the dogs fed the LY diet than those fed the other treatments. That microbial group has been reported to be more abundant in healthy dogs compared with those diagnosed with chronic enteropathy or small-cell lymphoma [48]. The *Ruminococcus gnavus* levels tended to be higher in the dogs consuming the MY and HY diets than those fed the control or LY diets. That bacterial group may support nutrient recycling and metabolism in healthy microbiota [49], although its overgrowth under dysbiotic conditions may contribute to disease states [50]. The relative abundance of *Peptoclostridium* was lower in the dogs fed the MY and HY diets than those fed the CTRL or LY diets. This bacterial genus has been previously associated with various disease conditions, including obesity and viral intestinal infections [51,52]. Although these findings may indicate microbial shifts in response to the treatments, limited information is available regarding the specific roles of this bacterial taxa in canine gut health, which limits the interpretation of their biological relevance.

Oxidative stress arises from an imbalance between the production and elimination of reactive oxygen species [53]. To maintain homeostasis, antioxidant systems and enzymes such as SOD neutralize superoxide, a major reactive oxygen species, by converting it into hydrogen peroxide, which is then further detoxified by catalase or glutathione peroxidase to prevent oxidative damage within the body [54]. Malondialdehyde, a byproduct of the lipid peroxidation of cell membranes, is also commonly used as a marker of oxidative stress [55]. Yeast-based ingredients have shown potential to mitigate oxidative stress by accumulating antioxidant vitamins and minerals and adsorbing phenolic compounds that enhance their antioxidant activity [2,3]. Studies in dogs suggest that yeast-derived supplements, such as spent yeast cell wall products, may reduce lipid peroxidation markers and increase the total antioxidant capacity, particularly under stress conditions [6]. In the current experiment, the greater post-transport MDA and SOD concentrations indicated that the stress condition (i.e., transport) elevated different oxidative stress markers. However, these markers were not different among the treatment groups. This lack of difference may be influenced by individual variability in oxidative responses, as dogs can exhibit differing physiological reactions to the same stressor. In this experiment, the changes (Δ) in MDA and SOD concentrations before and after transport averaged 5.04 ± 5.66 nmol/mL and 3.10 ± 9.40 ng/mL, respectively (mean ± SD). Additionally, the repeated exposure to the stress condition, as dictated by our experimental design, could have introduced additional confounding factors, limiting our ability to differentiate the treatment effects.

As expected, the serum chemistry and hematology measures were fairly consistent across treatments. Although some variation was observed in BUN, sodium, potassium, and chloride concentrations in the dogs fed the diets containing the yeast-enriched FCM, all values remained within the established reference ranges. Therefore, these results may have limited biological significance in healthy adult dogs. Nonetheless, future research in compromised or geriatric dog populations at risk for renal conditions should be considered.

To our knowledge, the effects of yeast-enriched FCM supplementation on biophysical parameters of the skin have not been previously investigated in dogs. In this experiment, the skin hydration status, sebum concentrations, and TEWL values were assessed as indicators of skin health in dogs. Adequate hydration is essential for maintaining the barrier function and to protect against external factors [56]. Similarly, TEWL measures moisture evaporation from the skin, with higher values indicating compromised barrier function and increased water loss, often linked to damaged or unhealthy skin [57,58]. Sebum supports the skin barrier function by reducing water loss, supporting thermoregulation, protecting against pathogens, and creating a glossy coat [7,59]. The supplementation of yeast-enriched FCM did not affect the sebum concentration, hydration status, or TEWL of the dogs. These results may be attributed to the fact that the dog population used in this study was in good health, which could have limited the potential for measurable improvements in the skin parameters we assessed. Therefore, further evaluation of the yeast-enriched FCM should be considered in compromised or challenged populations. Additionally, the methods used to measure the different skin parameters evaluated are standardized and validated for humans. However, given the differences in skin physiology between humans and dogs, the interpretation scales we used may not accurately differentiate optimal skin conditions in canines. The development of species-specific indices may be needed to ensure accurate assessments in future canine studies.

## 5. Conclusions

Yeast-enriched FCM may be effectively incorporated into extruded pet foods without compromising fecal characteristics, the ATTD of nutrients and energy, or skin measures. Although the responses were not large, extruded diets containing yeast-enriched FCM may influence the gut microbiota population and fermentation end-products. Although yeast-enriched FCM may possess antioxidant activity, no differences were observed in oxidative stress markers following transport stress, likely due to potential confounding factors inherent to our experimental design, which should be considered in future research. Additionally, higher yeast-enriched FCM inclusion levels may result in greater changes and may be of interest for further evaluation.

## Figures and Tables

**Table 1:** Ingredients and analyzed chemical compositions of the dietary treatments fed to the dogs.

	Treatments ^1^			
Item	CTRL	LY	MY	HY
Ingredients				
Chicken meal	25.0	25.0	25.0	25.0
Brewers rice	16.1	15.1	15.1	15.1
Corn	15.6	15.6	15.6	15.6
Sorghum	15.6	15.6	15.6	15.6
Corn gluten meal	7.5	7.5	7.5	7.5
Chicken fat	6.0	6.0	6.0	6.0
Dried egg product	5.0	5.0	5.0	5.0
Fish meal	2.5	2.5	2.5	2.5
Palatant	2.0	2.0	2.0	2.0
Beet pulp	1.5	1.5	1.5	1.5
Cellulose	1.5	1.5	1.5	1.5
FCM + LY	0.0	1.0	0.0	0.0
FCM + MY	0.0	0.0	1.0	0.0
FCM + HY	0.0	0.0	0.0	1.0
Monosodium phosphate hydrate	1.0	1.0	1.0	1.0
Choline chloride, 60%	0.3	0.3	0.3	0.3
Vitamin and mineral premix	0.25	0.25	0.25	0.25
Salmon oil	0.15	0.15	0.15	0.15
Taurine	0.08	0.08	0.08	0.08
	Chemical composition			
Dry matter (DM), %	92.11	92.16	90.36	91.94
	--- % DM ---			
Ash	6.30	6.26	6.49	6.61
Acid-hydrolyzed fat	14.12	12.77	13.84	12.62
Crude protein	35.90	32.79	34.44	33.33
Total dietary fiber	8.52	10.70	10.77	10.05
Gross energy ^2^, kcal/g	5.21	5.10	5.20	5.08

^1^ CTRL: control, FCM + LY: FCM + low yeast dose; FCM + MY: FCM + medium yeast dose; FCM + HY: FCM + high yeast dose. ^2^ Gross energy was measured via bomb calorimetry.

**Table 2:** Daily food and energy intake values of dogs and apparent total tract macronutrient digestibility of diets containing FCM enriched with different levels of dried yeast.

	Treatments ^1^					*p*-Value	
Item	CTRL	LY	MY	HY	SEM	TRT	C vs. T
Food intake (as-is), g/d	143.43	148.91	148.18	144.89	11.35	0.57	0.05
Food intake (DMB), g/d	132.49	138.68	135.30	134.67	11.11	0.68	0.23
Fecal output (as-is), g/d	289.22	344.92	337.35	321.55	30.56	0.07	0.01
Fecal output (DMB), g/d	104.96	122.77	124.56	119.95	10.21	0.08	<0.01
Digestibility, %							
Dry matter	84.16	82.35	82.26	82.41	0.78	0.24	0.04
Organic matter	87.05	85.48	85.15	85.56	0.65	0.16	0.02
Crude protein	84.34	82.54	83.38	83.01	0.87	0.43	0.13
Acid-hydrolyzed fat	94.04	92.62	93.31	92.75	0.47	0.06	0.01
Gross energy	87.49	86.42	86.07	85.97	0.65	0.30	0.06

^1^ CTRL: control; LY: FCM + low yeast dose; MY: FCM + medium yeast dose; HY: FCM + high yeast dose.

**Table 3:** Fecal characteristics and metabolite concentrations of dogs fed extruded diets containing FCM enriched with different levels of dried yeast.

	Treatments ^1^					*p*-Value	
Item	CTRL	LY	MY	HY	SEM	TRT	C vs. T
Fecal characteristics							
pH	6.37	6.73	6.78	6.65	0.19	0.39	0.10
Fecal dry matter (DM, %)	34.05	34.42	34.27	34.96	0.57	0.58	0.71
Fecal metabolites, µmol/g DM							
Total SCFA ^2^	499.75	567.20	496.30	514.73	0.62	0.07	0.30
Acetate	267.84	314.44	281.75	278.37	24.62	0.22	0.51
Propionate	158.10	163.75	135.14	139.16	14.98	0.10	0.83
Butyrate	73.81	89.00	79.41	97.20	10.45	0.45	0.28
Total BCFA ^3^	41.02	38.43	40.23	41.49	3.76	0.94	0.82
Isobutyrate	13.46	12.82	13.27	13.65	1.21	0.97	0.88
Isovalerate	25.41	23.22	24.59	25.24	2.33	0.91	0.69
Valerate	2.15	2.39	2.37	2.59	0.41	0.90	0.46
Total P/I ^4^	3.01 ^ab^	2.33 ^b^	3.35 ^a^	2.44 ^ab^	0.31	0.03	0.42
Phenol	0.58	0.43	0.40	0.35	0.09	0.30	0.09
Indole	2.43 ^ab^	1.90 ^b^	2.96 ^a^	2.10 ^ab^	0.28	0.03	0.71
Ammonia	85.14	84.02	87.44	87.60	6.19	0.97	0.87

^1^ CTRL: control; LY: FCM + low yeast dose; MY: FCM + medium yeast dose; HY: FCM + high yeast dose. ^2^ Total SCFA: total short-chain fatty acids = acetate + propionate + butyrate. ^3^ Total BCFA: total branched-chain fatty acids = isobutyrate + isovalerate + valerate. ^4^ Total P/I: total phenols and indoles. ^ab^ Means within a row with different superscripts differ (*p* < 0.05).

**Table 4:** Complete blood cell counts of dogs fed extruded diets containing FCM enriched with different levels of dried yeast.

		Treatments ^1^					*p*-Value	
Item ^2^	Reference Range	CTRL	LY	MY	HY	SEM	TRT	C vs. T
Red blood cells, 10^6^/µL	5.50–8.50	6.62	6.61	6.57	6.58	0.17	0.98	0.77
Hemoglobin, g/dL	12.0–18.0	15.41	15.33	15.25	15.19	0.37	0.92	0.50
Hematocrit, %	35.0–52.0	48.85	48.45	48.17	47.75	1.20	0.84	0.45
Mean cell volume, fl	58.0–76.0	74.02	73.50	73.18	72.57	1.04	0.94	0.77
MCH ^2^, pg	20.0–25.0	23.34	23.23	23.20	23.10	0.21	0.88	0.56
MCHC ^2^, g/dL	33.0–38.6	31.55	31.63	31.72	31.82	0.24	0.83	0.59
Platelets, 10^3^/µL	200–700	238.82	200.45	255.00	224.43	27.38	0.73	0.83
WBC ^2^, 10^3^/µL	6.0–17.0	4.74	4.54	4.74	4.69	0.60	0.38	0.23
Lymphocytes, 10^3^/µL	1.0–4.8	1.27	1.10	1.22	1.14	0.12	0.63	0.38
Monocytes, 10^3^/µL	0.2–1.4	0.12	0.19	0.15	0.26	0.06	0.26	0.58
Eosinophils, 10^3^/µL	0.1–1.0	0.18	0.13	0.15	0.15	0.05	0.09	0.03
Basophils, 10^3^/µL	0.0–2.0	0.02	0.00	0.01	0.01	0.002	0.11	0.02

^1^ CTRL: control; LY: FCM + low yeast dose; MY: FCM + medium yeast dose; HY: FCM + high yeast dose. ^2^ MCH, mean corpuscular hemoglobin; MCHC, mean corpuscular hemoglobin concentration; WBC, white blood cells.

**Table 5:** Serum chemistry profiles of dogs fed extruded diets containing FCM enriched with different levels of dried yeast.

		Treatments ^1^					*p*-Value	
Item ^1^	Reference Range	CTRL	LY	MY	HY	SEM	TRT	C vs. T
Creatinine, mg/dL	0.5–1.5	0.54	0.51	0.53	0.48	0.02	0.27	0.19
BUN ^2^, mg/dL	6–30	14.27 ^a^	13.55 ^ab^	14.55 ^a^	13.09 ^b^	0.78	<0.01	0.09
Total protein, g/dL	5.1–7.0	6.35	6.08	6.10	5.82	0.18	0.13	0.13
Albumin, g/dL	2.5–3.8	3.40 ^a^	3.27 ^ab^	3.35 ^ab^	3.14 ^b^	0.09	0.03	0.14
Globulin, g/dL	2.7–4.4	2.95	2.81	2.75	2.68	0.12	0.29	0.16
Albumin:globulin	0.6–1.1	1.17	1.17	1.25	1.18	0.05	0.12	0.35
Calcium, mg/dL	7.6–11.4	10.41	10.01	10.01	9.73	0.27	0.39	0.11
Phosphorus, mg/dL	2.7–5.2	3.46	3.34	3.25	3.21	0.18	0.45	0.19
Sodium, mmol/L	141–152	153.18	147.64	149.18	145.09	2.48	0.21	0.09
Potassium, mmol/L	3.9–5.5	4.34	4.13	4.19	4.14	0.08	0.14	0.03
Sodium:potassium	28–36	35.45	35.73	35.45	35.18	0.48	0.81	1.00
Chloride, mmol/L	107–118	117.91 ^a^	113.09 ^ab^	115.27 ^ab^	111.18 ^b^	1.96	<0.01	<0.01
Glucose, mg/dL	68–126	85.82	80.09	82.27	78.00	3.15	0.06	0.02
ALP ^2^, U/L	7–92	40.73	44.09	39.82	43.09	3.27	0.23	0.40
CALP ^2^, U/L	0–40	7.36	7.55	7.36	6.82	0.58	0.09	0.09
ALT ^2^, U/L	8–65	33.00	25.45	24.27	22.09	4.63	0.21	0.18
GGT ^2^, U/L	0–7	3.27	3.00	3.09	2.91	0.26	0.41	0.14
Total bilirubin, mg/dL	0.1–0.3	0.19	0.22	0.18	0.16	0.02	0.23	0.56
CPK ^2^, U/L	26–310	98.18	86.82	95.18	94.36	9.55	0.16	0.15
Cholesterol, mg/dL	129–297	218.18	210.45	217.27	202.73	8.99	0.13	0.26
Triglycerides, mg/dL	32–154	60.09	62.36	56.45	58.91	4.91	0.32	0.74

^1^ CTRL: control; LY: FCM + low yeast dose; MY: FCM + medium yeast dose; HY: FCM + high yeast dose. ^2^ BUN, blood urea nitrogen; ALP, total alkaline phosphatase; CALP, corticosteroid isozyme of ALP; ALT, alanine aminotransferase; GGT, gamma-glutamyltransferase; CPK, creatinine phosphokinase. ^ab^ Means within a row with different superscripts differ (*p* < 0.05).

**Table 6:** Serum superoxide dismutase (SOD) and malondialdehyde (MDA) concentrations of dogs fed extruded diets containing FCM enriched with different levels of dried yeast, measured before and after transport.

	Treatments ^1^					Time			*p*-Value			
Item	CTRL	LY	MY	HY	SEM	Pre	Post	SEM	TRT	TIME	INT	C vs. T
SOD, ng/mL	31.94	30.43	30.84	29.52	4.96	29.14	32.23	10.6	0.92	0.06	0.81	0.54
MDA, nmol/mL	16.61	15.71	15.51	14.87	1.04	13.15 ^b^	18.20 ^a^	0.88	0.63	<0.01	0.16	0.30

^1^ CTRL: control; LY: FCM + low yeast dose; MY: FCM + medium yeast dose; HY: FCM + high yeast dose. ^ab^ Means within a row with different superscripts differ (*p* < 0.05).

**Table 7:** Sebum concentration, hydration status, and transepidermal water loss (TEWL) values of dogs fed extruded diets containing FCM enriched with different levels of dried yeast.

	Treatments ^1^					*p*-Value	
Item	CTRL	LY	MY	HY	SEM	TRT	C vs. T
Sebum concentration ^2^, arbitrary unit							
Back	1.55	1.76	1.73	1.97	0.49	0.92	0.59
Inguinal	3.03	3.21	3.58	2.52	1.33	0.91	0.62
Ear	19.76	25.61	28.18	24.27	4.67	0.58	0.22
Hydration ^3^, arbitrary unit							
Back	0.06	0.03	0.21	0.00	0.07	0.21	0.73
Inguinal	10.06	11.88	13.73	11.42	1.62	0.42	0.21
Ear	7.48	9.39	9.61	8.30	1.74	0.79	0.41
TEWL ^4^, g/h/m^2^							
Back	10.16	9.88	10.51	9.45	1.12	0.92	0.87
Inguinal	8.70	9.66	9.47	9.12	0.50	0.55	0.22
Ear	8.27	7.94	7.90	7.74	0.47	0.85	0.41

^1^ CTRL: control; LY: FCM + low yeast dose; MY: FCM + medium yeast dose; HY: FCM + high yeast dose. ^2^ Measured using a sebumeter, μg/cm^2^. ^3^ Measured using a corneometer, arbitrary units. ^4^ Measured using a tewameter, g/h/m^2^.

**Table 8:** Relative abundance levels (% of sequences) of predominant bacterial genera in feces of dogs fed extruded diets containing FCM enriched with different levels of dried yeast.

	Treatments ^1^					*p*-Value	
Taxa	CTRL	LY	MY	HY	SEM	TRT	C vs. T
Actinobacteriota							
*Bifidobacterium*	1.10	1.55	0.72	2.01	0.63	0.44	0.75
Bacteroidota							
*Alloprevotella*	2.65	2.18	2.88	1.93	0.58	0.73	0.89
*Bacteroides*	6.39	8.74	8.27	6.45	1.47	0.26	0.24
*Muribaculaceae*	0.43	1.21	1.60	0.90	0.53	0.26	0.13
*Prevotella*	1.50	2.19	2.19	2.33	0.58	0.55	0.16
Firmicutes							
*Allobaculum*	3.98	7.07	7.07	8.05	0.47	0.42	0.14
*Blautia*	3.95	3.63	3.62	3.50	0.51	0.88	0.44
*Eubacterium brachy group*	1.07 ^ab^	0.71 ^b^	1.24 ^a^	1.28 ^a^	0.24	0.04	0.57
*Faecalibacterium*	3.74	3.13	3.76	3.04	0.76	0.73	0.46
*Lactobacillus*	11.44	10.03	8.63	13.76	3.05	0.44	0.93
*Megamonas*	1.50	2.09	1.02	1.22	0.48	0.13	0.88
*Peptoclostridium*	12.97	10.08	9.34	10.93	1.22	0.10	0.02
*Phascolarctobacterium*	0.94	1.15	1.42	1.00	0.21	0.32	0.28
*Romboutsia*	0.99	1.32	1.05	1.14	0.36	0.85	0.52
*Ruminococcus gnavus group*	1.37	1.25	0.80	0.81	0.28	0.09	0.04
*Ruminococcus torques group*	1.20	0.87	1.06	1.05	0.18	0.40	0.20
*Turicibacter*	2.74	2.92	1.81	2.28	1.00	0.81	0.37
Fusobacteriota							
*Fusobacterium*	16.56	16.63	20.38	15.73	2.41	0.34	0.65
Proteobacteria							
*Anaerobiospirillum*	1.55	1.26	0.65	0.58	0.41	0.56	0.19
*Parasutterella*	2.02	2.27	2.30	2.14	0.88	0.72	0.66
*Sutterella*	1.23	1.45	2.00	1.29	0.65	0.53	0.33

^1^ CTRL: control; LY: FCM + low yeast dose; MY: FCM + medium yeast dose; HY: FCM + high yeast dose. ^ab^ Means within a row with different superscripts differ (*p* < 0.05).

## Data Availability

The data that support the findings of this experiment are available from the corresponding author upon reasonable request. These data are not publicly available due to their commercial value.

## Supplementary Materials

The following supporting information can be downloaded at https://www.mdpi.com/article/10.3390/ani15071046/s1: Figure S1. Fecal score distribution in dogs fed extruded diets containing FCM enriched with different levels of dried yeast. CTRL = control, LY = FCM + low yeast dose, MY = FCM + medium yeast dose, HY = FCM + high yeast dose.

## Abbreviations

ATTD, apparent total tract digestibility; BCFA, branched-chain fatty acids; DM, dry matter; FCM, functionalized canola meal; HY, diet containing FCM + high yeast dose; LY, diet containing FCM + low yeast dose; MDA, malondialdehyde; MOS, mannanoligosaccharides; MY, diet containing FCM + medium yeast dose; SCFA, short-chain fatty acids, SOD, superoxide dismutase; TDF, total dietary fiber; TEWL, transepidermal water loss; UniFrac, unique fraction metric
