# Supplementary material for: Effects of Yeast Products on the Apparent Total Tract Macronutrient Digestibility, Oxidative Stress Markers, Skin Measures, and Fecal Characteristics and Microbiota Populations of Healthy Adult Dogs
Source: Animals (Basel). 2025 Apr 4;15(7):1046. doi: 10.3390/ani15071046 (PMC11987801; doi:10.3390/ani15071046)

Figure S1: Fecal score distribution in dogs fed extruded diets containing FCM enriched with different levels of dried yeast. CTRL = control, LY = FCM + low yeast dose, MY = FCM + medium yeast dose, HY= FCM + high yeast dose.

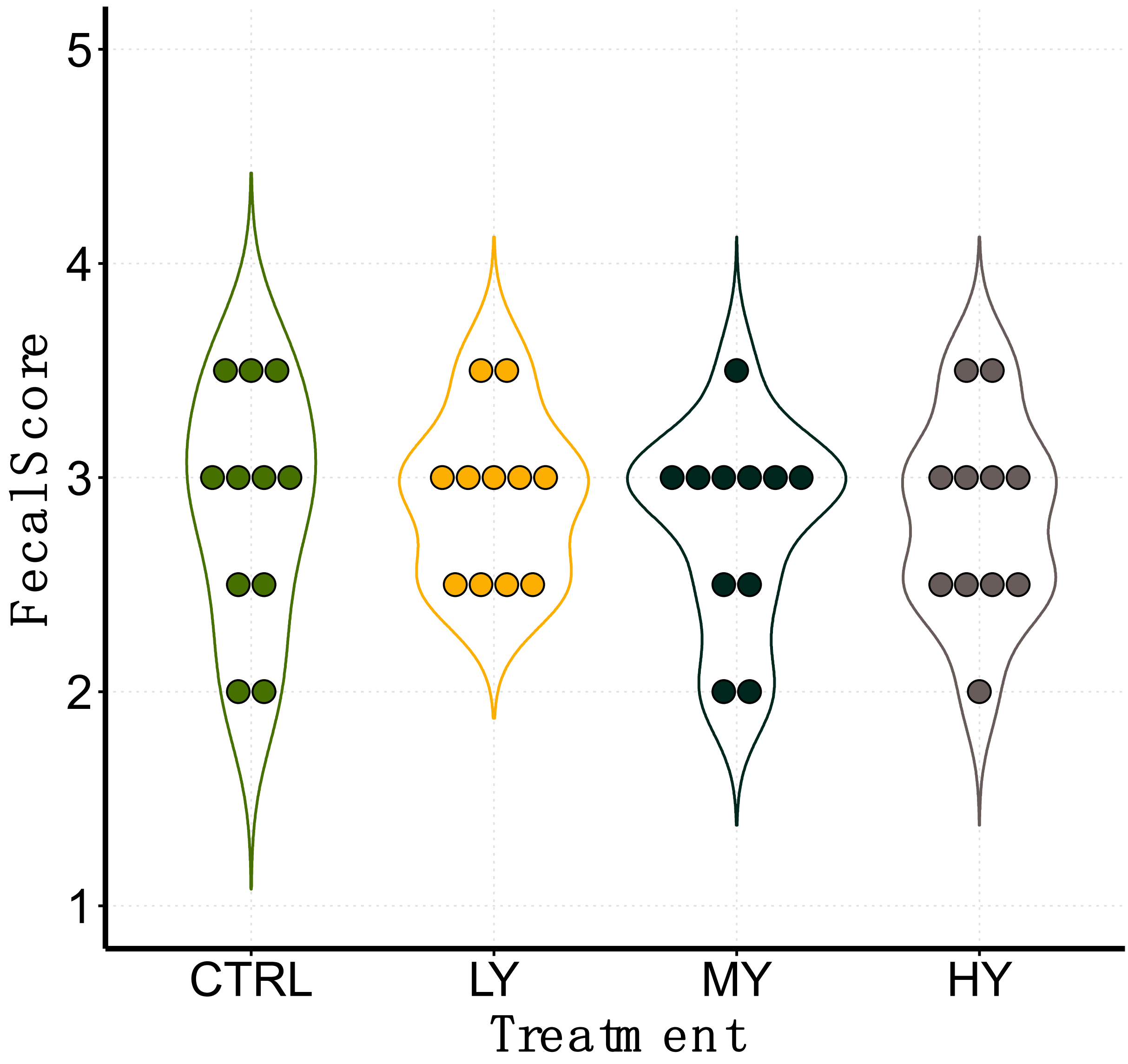

Supplement: Supplementary file 1 [file animals-15-01046-s001.zip › animals-3558084-supplementary.pdf]
